# The expanding role of aerosols in systemic drug delivery, gene therapy and vaccination: an update

**DOI:** 10.1186/2213-0802-2-3

**Published:** 2014-01-13

**Authors:** Beth L Laube

**Affiliations:** The Johns Hopkins Medical Institutions, Suite 3015, The David M. Rubenstein Building, 200 North Wolfe Street, Baltimore, MD 21287 USA

**Keywords:** Systemic drug delivery by inhalation, Aerosolized gene therapy, Vaccination by inhalation

## Abstract

Until the late 1990s, aerosol therapy consisted of beta_2_-adrenergic agonists, anti-cholinergics, steroidal and non-steroidal agents, mucolytics and antibiotics that were used to treat patients with asthma, COPD and cystic fibrosis. Since then, inhalation therapy has matured to include drugs that: (1) are designed to treat diseases outside the lung and whose target is the systemic circulation (systemic drug delivery); (2) deliver nucleic acids that lead to permanent expression of a gene construct, or protein coding sequence, in a population of cells (gene therapy); and (3) provide needle-free immunization against disease (aerosolized vaccination). During the evolution of these advanced applications, it was also necessary to develop new devices that provided increased dosing efficiency and less loss during delivery. This review will present an update on the success of each of these new applications and their devices. The early promise of aerosolized systemic drug delivery and its outlook for future success will be highlighted. In addition, the challenges to aerosolized gene therapy and the need for appropriate gene vectors will be discussed. Finally, progress in the development of aerosolized vaccination will be presented. The continued expansion of the role of aerosol therapy in the future will depend on: (1) improving the bioavailability of systemically delivered drugs; (2) developing gene therapy vectors that can efficiently penetrate the mucus barrier and cell membrane, navigate the cell cytoplasm and efficiently transfer DNA material to the cell nucleus; (3) improving delivery of gene vectors and vaccines to infants; and (4) developing formulations that are safe for acute and chronic administrations.

## Introduction

There are many advantages to administering medications to the lung as an aerosol. These include: a more rapid onset of action for short-acting bronchodilators, compared to oral therapy; high local concentration by delivery directly to the airways; needle-free systemic delivery of drugs with poor oral bioavailability; and pain- and needle-free delivery for drugs that require subcutaneous or intravenous injection. Traditional aerosol therapies with the lung as the target consist of short-acting β_2_-adrenergic agonists and long-acting β_2_-adrenergic agonists (LABA), anticholinergics, inhaled corticosteroids (ICSs), nonsteroidal anti-inflammatories, antibiotics and mucolytics. Devices that are available to deliver these drugs include pressurized metered-dose inhalers (pMDIs), used either alone, or attached to spacers, or valved holding chambers (VHCs), breathactuated (BA)-pMDIs, dry powder inhalers (DPIs), jet nebulizers, vibrating mesh nebulizers and soft mist inhalers. Well-established treatment guidelines for the management of asthma [1] and chronic obstructive pulmonary disease (COPD) [2] each recommend inhaled therapy as the primary route to administer these medications. Treatment guidelines for cystic fibrosis (CF) also include recommendations for inhalation of aerosolized medications [3, 4]. Guidelines for inhalation therapy to treat these diseases will not be covered in this review. A comprehensive presentation of old and newly-approved devices and their correct use in treating these diseases has also been published [5] and will not be included in this review. This review will focus on the newest applications for aerosol therapy by oral inhalation. When possible, results from clinical studies, rather than preclinical studies, will be highlighted and delivery devices will be included if they are a new design and critical to the success of the application. Updates on new applications for intranasal therapy are beyond the scope of this review and can be found in several referenced articles [6–14].

New applications for oral inhalation now include drugs that: (1) target the systemic circulation as a means to treat disorders unrelated to the lung (systemic drug delivery by inhalation); (2) deliver nucleic acids that lead to permanent expression of a gene construct, or protein coding sequence, in a population of cells within the lung, thereby reversing or preventing a disease process (aerosolized gene therapy); and (3) provide needle-free immunization and prevention against infectious diseases (vaccination by inhalation). Since first reviewing this subject in 2005 [15], each of these new applications has met with varying degrees of success in terms of achieving clinical efficacy and commercialization. This brief review provides an update on the status and challenges facing each of these new applications and focuses on an example that is furthest along in development and/or affects the most people. Within each example are success stories, failures and lessons to be learned. Addressing those lessons will enhance these applications in the future.

## Review

### Systemic drug delivery by inhalation

Because of the many advantages to aerosol therapy mentioned above, a number of systemically active drugs have been developed as possible candidates for aerosol delivery through the lung into the systemic circulation. For these drugs, it is important that delivery lead to adequate systemic absorption with no irritability, or damage, to the airways or alveoli. Drugs that have been tested include opioids for pre- and post-operative analgesia, dihydroergotamine (DHE) for acute treatment of migraine, interferon β to treat multiple sclerosis, leuprolide acetate to treat prostatic cancer, infertility and post-menopausal breast cancer, calcitonin to treat postmenopausal osteoporosis, growth hormone releasing factor to treat pituitary dwarfism and insulin to treat diabetes.

A few of these drugs have shown promise in human trials and some have been commercialized. Opioids such as morphine and fentanyl have been tested as a liquid aerosol generated by traditional jet nebulizers [16] and by the AERx^®^ vibrating mesh prototype nebulizer [17]. The usefulness of inhaled opioids lies in the elimination of an intravenous catheter with the potential of providing rapid-onset, patient-controlled analgesia. A large variability in absorption was reported with jet nebulizer administration [16], which likely will limit acceptance as a method for pre- or post-operative pain control with these devices. Pain management was more predictable with the AERx^®^ device (Aradigm Corp., Hayward, CA, USA) [17]. Another pain management drug, DHE, has proven superior to placebo for the acute treatment of migraine in a phase 3, double-blinded, multicenter study [18]. In that study, a novel formulation of DHE (LEVADEX™, MAP Pharmaceuticals, Mountain View, CA, USA) was delivered to the systemic circulation using the TEMPO^®^ pMDI (MAP Pharmaceuticals). Calcitonin has been successfully delivered to the systemic circulation intranasally [7, 8] and is now available as Miacalcin^®^ (Novartis Pharmaceuticals Corp, East Hanover, NJ, USA) nasal spray to treat postmenopausal osteoporosis in females greater than 5 years post menopause.

#### Best example of systemic drug delivery by aerosolization: treating diabetes with oral inhalation of insulin

Although there are several other successful drugs that have been administered to the systemic circulation by inhalation, the best example of the expanding role of aerosol therapy into systemic drug delivery is treating diabetes with oral inhalation of insulin. This is because this route of administration has the potential to eliminate subcutaneous (SC) injection of insulin for a significantly large patient population. An estimated 370 million people worldwide have diabetes [19] and the majority of these have non-insulin dependent diabetes mellitus (NIDDM), or type-2 diabetes. The goal for the treatment of type-2 diabetes is to maintain glucose control in the normal range to prevent long-term complications. Typically, the first line of treatment is oral anti-diabetic medications. Eventually, anti-diabetic medications fail and type-2 patients need to administer insulin SC four times/day (i.e. before meals and at bedtime) to achieve good control. Because injection hurts, compliance with treatment is often reduced. More importantly, patients who would benefit from early intervention with insulin treatment decline treatment because of the pain and inconvenience associated with injection.

A second reason why treating diabetes with oral inhalation of insulin is the best example of the expanding role of aerosol therapy into systemic drug delivery is because what we now know about systemic drug delivery of peptides by inhalation was learned during the development of inhaled insulin and the study of its success and early failure continues to inform future development of systemic drug delivery by inhalation, as well as other new applications of aerosol therapy.

The notion that insulin could be administered through the lung to the systemic circulation by inhalation was investigated in the 1970s [20, 21]. From those early trials, several challenges were identified that needed to be overcome. These included: (1) determining the appropriate lung target; (2) determining the inhaled dose that controls blood glucose levels; and (3) developing new devices that deliver the dose to the target. By the early 1990’s, several pharmaceutical companies and device manufacturers began to address these challenges. Work in animals showed that the appropriate target for aerosolized insulin and other drugs delivered through the lung for systemic administration is the alveolar region [22]. This is because the alveolar region comprises a resorptive surface of 50-75 m^2^, which provides a surface area for drug absorption that is the size of a tennis court. In addition, mucociliary clearance is minimal in the alveolar region. Once drug deposits in the alveolar region, the residence time is long, enhancing the probability of absorption. Finally, the cell barrier to absorption is extremely thin (0.1 mm) in the alveolar region, thereby enhancing the possibility for absorption from the epithelial layer to the lung vasculature.

By the late 1990s, results from several studies [23–26] showed that a dose of 1.0 U/kg body weight human regular insulin aerosol controlled fasting glucose levels and a dose of 1.5 U/kg body weight controlled postprandial glucose levels. However, this dose posed an early limitation to this route of administration, since it was approximately 10 times the dose given subcutaneously (i.e. ~0.1 U/kg body weight).

Nevertheless, the Exubera^®^ Pulmonary Insulin Delivery System (Pfizer Pharmaceuticals, New York, NY; and Nektar Therapeutics, San Carlos, CA), was approved by the U.S. Food and Drug Administration in 2006 for use in adults with both type 1 and type 2 diabetes and became commercially available soon thereafter [27, 28]. By early 2007, two additional devices and formulations were in Phase III testing. These included the AERx^®^ Insulin Diabetes Management System (Novo-Nordisk A/S, Bagsverd, Denmark; and Aradigm Corp., Hayward, CA) and the AIR^®^ Inhaled Insulin System (Eli Lilly and Co., Indianapolis, IN; and Alkermes Inc., Cambridge, MA) [29–31]. However, between October, 2007 and May, 2008, production of all three products had been discontinued.

#### What went wrong?

1. The cost of inhaled insulin was higher than injectable insulin. One analysis demonstrated that Exubera^®^’s inhaled insulin cost about $5 per day, compared to $2-3 per day by injection [32]. The higher cost was due in part to the lower bioavailability of Exubera^®^ inhaled insulin compared to SC. The bioavailability of Exubera was only 10-15% of the SC dose [26].
2. Safety became an issue. Studies with Exubera^®^ that lasted 6 months, showed increased insulin binding antibodies, coughing and a reduction in diffusing capacity, compared to injected insulin [33]. However, longer-term studies showed that reductions in lung function parameters were small, non-progressive and reversible with discontinuation of treatment [33].
3. Sales in the U.S. of Exubera^®^ were lower than expected, perhaps because of the cost, or safety issues, or because few patients, or care givers, realized the advantages of this route of administration compared to injection therapy.

#### Second generation delivery system for inhaled insulin

**Figure 1:**
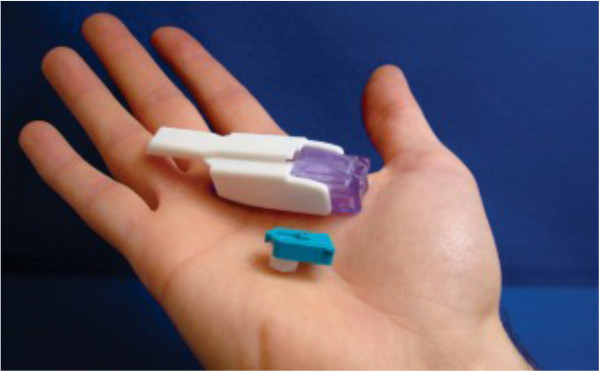
**The AFREZZA** ^**®**^ **(MannKind Corporation, Valencia, CA, USA): a second generation device for delivering dry powder insulin.** It is a drug-device combination product, consisting of AFREZZA inhalation powder pre-metered into single use dose cartridges and a lightweight, AFREZZA inhaler. Insulin is placed into the chamber in an aspirin-like tablet. Closing the device crushes it into a fine powder which is then inhaled by the patient (downloaded from MannKind website at http://www.mannkindcorp.com/product-pipeline-diabetes-afrezza.htm).

**Figure 2:**
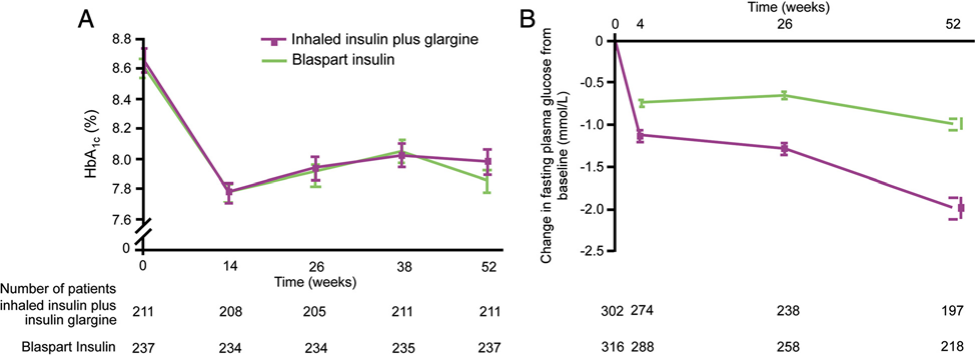
**Results from recent clinical trial with inhaled Technosphere insulin plus insulin glargine.** **(A)** Glycosylated hemoglobin (HbA_1c_) values over 52 weeks for patients who were treated with inhaled Technosphere insulin plus insulin glargine versus patients who were treated with Biaspart insulin by SC; **(B)** Change in fasting plasma glucose from baseline for two patient groups over 52 weeks (from Reference [36] with Permission).

AFREZZA^®^ is a pocket-size device developed by MannKind Corporation (Valencia, CA, USA) (Figure 1) [34]. It is a second generation delivery system for inhaled insulin with several advantages over earlier generations. First, it delivers microparticles (Technospheres™) of insulin. Technosphere™ insulin particles (human regular insulin loaded onto a fumaryl diketopiperazine molecule) are optimized for deposition in the alveolar region of the lung. Greater than 90% of the particles are in the respirable range, with a mean particle diameter of 2.5 μm [35]. Bioavailability of this new formulation is also estimated to be 24-28% of SC [36] which is higher than for human regular insulin delivered by the Exubera^®^ device. Unlike the Exubera^®^, it is small and portable and appears to be easier to use. In a recent clinical trial, the change in HBA_1C_ for 211 type 2 patients after 52 weeks on prandial inhaled Technosphere plus bedtime insulin glargine was similar and non-inferior to that of 237 patients who were injected twice daily with biaspart insulin (70% insulin aspart protamine suspension and 30% insulin aspart) (Figure 2A and 2B) [36]. In addition, weight gain was lower and hypoglycemic events were fewer on Technoshpere compared to patients on injected insulin. Thirty-two percent of patients treated with Technosphere reported cough compared to 14% of patients treated with injected insulin. Most cough events occurred during the first 10 minutes of inhalation and declined to about 2/week by week 6. There were no differences between treatment groups in terms of pulmonary function changes. A recent review based on a MEDLINE search of studies relevant to Technosphere insulin concludes that it is has a pharmacokinetic profile suitable to meet prandial insulin needs in patients with diabetes [37]. The company is awaiting FDA approval for mealtime glucose control only. If approved, it could lead to earlier treatment of diabetes with insulin for patients who have resisted such treatment due to fear of, or pain associated with, injection therapy.

#### Future directions

To insure satisfactory outcomes and patient acceptability of systemic drug delivery by aerosolization in the future, it is clear that the bioavailability of expensive drugs like insulin with relatively low bioavailability needs to be improved. Suggestions for improving bioavailability include: better targeting of the alveolar region with nanoparticle (<0.1 μm in diameter) formulations, or formulations containing porous particles that have aerodynamic characteristics similar to extrafine particles (~1.0 μm in diameter); and enhancing absorption by adding absorption enhancers that do not damage lung tissue. Several additives that have been tested in rats appear to enhance absorption and permeation and may be appropriate to improve absorption in future pulmonary protein formulations. These include endogenous surfactants such as DPPC [38, 39], citric acid [40], and hydroxypropylcellulose [41].

It is also clear that: (1) formulations are needed that do not produce cough, or changes in lung function, and are safe for acute and chronic administrations; (2) the device should be small, portable and easy to use; (3) the total cost of the device and formulation should be similar in cost to the injection product; and (4) patients and physicians should be well-educated in terms of the advantages of this route of a dministration compared to injection therapy to ensure compliance.

### Aerosolized gene therapy

The lung is an important target organ for gene therapy. This is because there are a number of lung diseases that could benefit from this type of treatment. These include: lung cancer, asthma, cystic fibrosis and alpha-1-antitrypsin deficiency. The goal of aerosolized gene therapy is to correct the lung disorder with delivery of a functional copy of the aberrant gene to the appropriate target within the lung.

#### Viral vectors versus non-viral vectors for gene therapy

As with systemic drug delivery by aerosolization, aerosolized gene therapy will likely require repeat dosing. In order to avoid lung damage as a result of repeat therapy, it is important that the vector that is selected to deliver the functional genetic material does not cause immune responses from the host, or lead to mutagenesis over time. Although there are a number of viral and non-viral vectors that are available for aerosolization that can deliver the functional genetic material, the identification of safe vectors has been one of the major challenges to the development of aerosolized gene therapy [42, 43]. Viral vectors include retroviruses, lentiviruses, adenoviruses (Ad) and adeno-associated viruses (AAV). Retroviruses are capable of long-term gene expression following genomic integration but only in non-dividing cells, whereas, lentiviruses are capable of long-term gene expression in both non-dividing and dividing cells. However, both types of viruses present a high risk of insertional mutation leading to oncogenesis. Adenoviruses infect non-replicating cells, show potential for persistent expression and present low risk of insertional mutagenesis. However, they can trigger a strong immune response by the host. Unlike adenoviruses, AAV do not trigger a strong immune response by the host, but they have a small packaging capability (~4.7 kb), so they are limited in the amount of genetic material they can carry, and they have reduced efficacy with repeat-administration.

Non-viral vectors are carrier molecules that are either cationic lipids, or cationic polymers, that bind to negatively charged plasmid DNA and either encapsulate or condense the DNA to generate lipoplexes and polyplexes. Non-viral vectors are generally less efficient than viral vectors because they lack specific components that could help with endosomal escape, movement through the cytoplasm and nuclear uptake. The simpler composition of non-viral vectors, however, may have an advantage over viral vectors, since they are free of non-human components, making re-administration potentially more successful [44, 45].

Of the vectors available for delivering genetic material for gene therapy, AAV vectors have been the most utilized in terms of animal experiments and clinical trials. Currently, the most common carrier is the viral vector AAV2, but recent studies suggest that AAV2 with capsids from serotypes 1, 5 and 6 may be more efficient in transducing airway epithelial cells than AAV2 [46, 47]. Some progress has been made in improving non-viral gene transfer [48], but more safety data is needed before they can be safely administered to humans on a chronic basis. There are currently no dry-powder, or metered-dose inhaler formulations for any vector-drug combination. Therefore, the field is further limited by delivery in a liquid formulation using a nebulizer.

#### Best example of aerosolized gene therapy: treating cystic fibrosis (CF)

Development of aerosolized gene therapy for lung cancer and alpha-1-antitrypsin deficiency has not progressed beyond the preclinical stage. Results from clinical trials with early therapies have not shown efficacy. On the other hand, aerosolized gene therapy for treating cystic fibrosis has had some success in clinical trials and details of those efforts are reported here. Approximately 70,000 people worldwide have cystic fibrosis (CF), an inherited, autosomal recessive disease. Mutations in the cystic fibrosis transmembrane conductance regulator (CFTR) gene lead to loss of chloride, sodium and water transport, impaired mucus removal, obstructed airways, chronic infection and end stage lung disease. The goal of aerosolized gene therapy in treating cystic fibrosis is to restore CFTR function and normal chloride channel function in the lungs.

Over the years, there have been a number of challenges to aerosol delivery of vectors carrying intact CFTR complementary DNA (cDNA). First, there has been the challenge of delivering an adequate dose to infants, who are an important target population. This is because identification of infants who are afflicted with CF is now possible at birth and early gene therapy holds the promise of correcting the abnormality before irreversible lung damage can occur. However, due to anatomic, physiologic and behavioral factors, delivery of aerosols to infants is challenging and highly variable. Gene transfer therapy is likely to be extremely expensive, so improving delivery efficiency and reducing variability of delivery to small children will result in less waste and help insure the desired effect.

**Figure 3:**
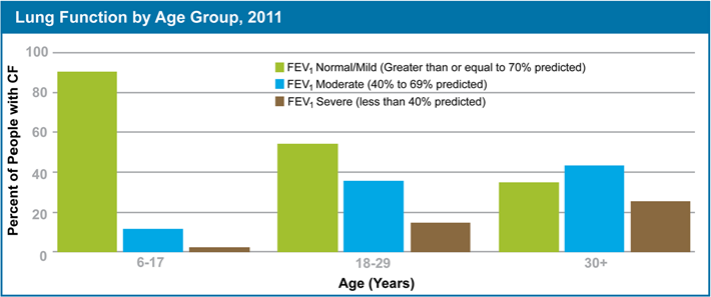
**Percent of people with cystic fibrosis by age with normal/mild forced expiratory volume in one second (FEV**_**1**_ **), moderate FEV**_**1**_ **and severe FEV**_**1**_ **.** The majority of children have FEV_1_ values in the normal to mild range, indicating mild disease and mild airway obstruction. Adults age 18–29 and 30+ have FEV_1_ values in the moderate to severe range, indicating severe disease and increased airway obstruction (downloaded from Cystic Fibrosis Foundation website at http://www.cff.org/UploadedFiles/Research/ClinicalResearch/2011-Patient-Registry.pdf).

**Figure 4:**
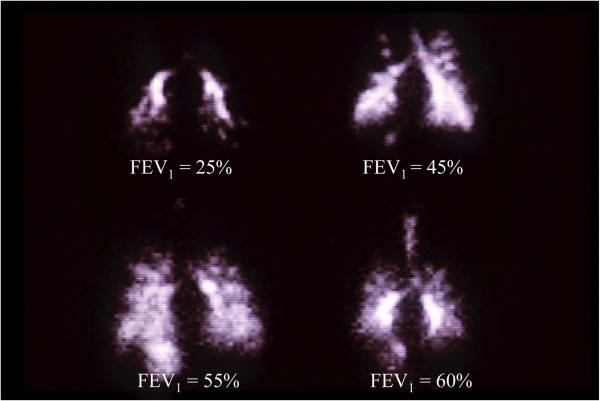
**Gamma camera images of four adult patients with cystic fibrosis and different FEV**_**1**_ **values showing distribution of an aerosol containing the radioisotope** ^**99m**^ **technetium.** Uniform deposition of aerosolized radioisotope is reduced in patients with low FEV_1_ and severe obstruction (from Reference [15] with Permission).

Another challenge has been uniform delivery of the drug vector to the lungs of adult patients with CF. Disease in adults with CF is significantly more severe, compared to children with CF (Figure 3) [49]. Increased disease severity is shown by a lower percent forced expiratory volume in one second (FEV_1_) in adults, compared to children. Increased severity in disease leads to uneven distribution of the drug vector, with areas of the lung that are unobstructed receiving a higher dose of vector than regions that are partially, or fully obstructed (Figure 4). Such uneven distribution of the drug vector could make treatment less efficacious. These same challenges are likely to apply to treating infants, or adults with obstructed airways, with aerosolized gene vectors for other lung diseases.

Additional challenges to aerosolized gene therapy for CF that likely apply to aerosolized gene therapy for other lung diseases include delivery of DNA through the mucus barrier. The mucus barrier is thick and viscous in patients with CF, and in patients with chronic obstructive lung disease (COPD), there is excess mucus production. Gene vectors whose target is the airway epithelial cell must be able to penetrate beyond the mucus barrier to reach their cell target. Another challenge is the need for vectors that recognize receptors on the apical surface of airway epithelial cells. Many vectors only recognize receptors on the basal-lateral surfaces, which are very difficult to access. The gene vector must deliver DNA to the cell nucleus. To do this, the vector must penetrate the cytoplasmic membrane, overcome cytoplasmic proteases and penetrate the nuclear membrane.

To date, 24 clinical trials with aerosolized gene vectors have been carried out since the cloning of the CF gene in 1989. Nine Ad CF gene therapy trials were carried out in the upper and lower airways of CF patients between 1993 and 2001 [50]. These trials showed that low level gene transfer can be achieved in some patients, but administration resulted in lung inflammation and induced humoral and cellular immune responses, affecting the efficacy of re-administration. These shortcomings have not been overcome [48].

Between 1999 and 2007, six clinical trials were carried out with the AAV2 serotype [51]. Acute administration appeared to be safe, but assessment of the efficiency of vector-specific expression was lacking and in another large trial there was no improvement in lung function [52]. Moreover, repeat dosing with the AAV2 serotype does not appear to be possible due to the development of an anti-viral immune response.

Nine clinical trials have evaluated non-viral gene transfer to the upper and lower airways of CF patients [50]. For the most part these were proof of principle and Phase I safety studies. None of the trials were designed to assess clinical efficacy. A recent review article provides detailed information about these clinical trials, what vectors were used and their drawbacks [45].

Recent developments in the UK hold new promise for improving CF lung disease through gene therapy [45]. The UK CF Gene Therapy Consortium (http://www.cfgenetherapy.org.uk) is currently conducting the only active CF gene therapy clinical trial. This will be a multi-dose clinical trial using the non-viral cationic lipid formulation GL67A (Genzyme, Cambridge, MA, USA) with certain modifications, including CpG-depletion and the incorporation of an hCEFI promoter called pGM169 [45]. CpG-free plasmids reduce inflammation and lead to long-acting gene expression when administered to the mouse lung [45, 53]. The hCEFI promoter also prolongs gene expression in mice [53]. A safe dose for a multi-dose double-blinded placebo-controlled trial of this new formulation has been determined and trial participants have been recruited [54]. Participants will be treated with 12 monthly doses delivered by the AeroEclipseII Breath-Actuated Nebulizer (Trudell Medical Instruments, London, Canada) over a year period [55].

#### Future directions

Successful correction of lung diseases with inhaled gene therapy remains elusive. A number of challenges must be overcome before pulmonary gene therapy becomes a reality. These include: (1) developing gene vectors that can more efficiently penetrate the mucus barrier and cell membrane, navigate the cell cytoplasm and transfer DNA material to the cell nucleus; (2) improving delivery of gene vectors to infants; and (3) developing formulations that are safe and effective for acute and chronic administrations.

### Vaccination by inhalation

The rationale for aerosolized vaccination is based on the following advantages over injection therapy, particularly in developing countries. First, vaccination by inhalation avoids the need for disposal strategies for the large number of needles that are used in mass campaigns in developing countries. Secondly, it prevents the spread of blood borne diseases such as hepatitis B and HIV, which can be transmitted by improper use and handling of used sharps. Thirdly, administration of a vaccine via the aerosol route has less need for medical personnel, or a medical setting, compared to administration by injection, and should facilitate vaccination implementation in developing countries. Another rationale for aerosolized vaccination is that it induces protection by exposure of the airway mucosa to agents that directly affect the lungs and cause diseases such as tuberculosis, diphtheria, pneumococcal pneumonia, measles, mumps and rubella. Airway mucosal vaccination may also represent a potential approach for immunizing against agents that do not directly affect the lungs such as human papilloma virus, or hepatitis B virus, by inducing relevant antibodies in the serum.

Because of the advantages to aerosolized immunization, a number of vaccines are being tested for feasibility and efficacy via the pulmonary route. Many of these are still in preclinical stages and have not progressed to clinical trials. Brief descriptions of results from these early studies are described here. Hepatitis B virus infection remains an important global health concern despite effective vaccines that are available by injection. But, for reasons mentioned above, injection therapy for hepatitis B is restricted in the developing world. Although inhalation therapy is of interest, it is unknown if immunization is possible by inhalation of hepatitis vaccine. A few animal studies have begun to investigate the effects of particle size and formulation on this route of administration. A liquid suspension of hepatitis B vaccine PLGA (poly-D,L-lactide-co-glycolide) or PLA (poly-lactic acid), nanoparticles of different sizes administered to rats using a Microsprayer^®^ (Penn-Century, Inc., Wyndmoor, PA, USA) resulted in humoral and mucosal immune responses that varied with particle size and hydrophobicity of the polymers used [56]. A dry powder of hepatitis B vaccine nanoparticles administered by the Insufflator^®^ (Penn-Century, Inc.) led to lower IgG and higher IgA in guinea pigs, compared to intramuscular injection [57]. A liquid suspension of PLGA microspheres of hepatitis B vaccine, administered by Microsprayer^®^, showed that immunogenicity in rats was a function of particle size [58].

Safety and tolerance of intranasal administration of hepatitis B vaccine (NASVAC), comprised of hepatitis B virus (HBV) surface (HBsAg) and core antigens (HBcAg), with Accuspray^®^ (Becton Dickinson and Company, Franklin Lakes NJ, USA) has been demonstrated in a small group of healthy volunteers [59]. However, large, randomized controlled clinical trials with this inhaled hepatitis B vaccine are needed to show efficacy.

Administration of diphtheria vaccine by inhalation is of interest because it would avoid the high probability of a local reaction that occurs at the site of vaccination with intramuscular injection. It would be a safer route of administration in developing countries and might induce mucosal IgA antibody which could bind to the exotoxin released by *Cornyebacterium diphtheriae,* preventing it from entering and colonizing the airway mucosal membrane [60]. Inhaled diphtheria vaccine is in early stages of development, but a dry powder formulation of diphtheria CRM-197 antigen with PLGA as an adjuvant, administered using the Insufflator^®^, resulted in lower IgG in the sera and higher IgA in the BAL of guinea pigs, compared to intramuscular injection [60].

The urgency to address drug-resistant TB has led to a resurgence in interest in inhalation as a route of administration for anti-TB drugs to treat TB as well as vaccines to prevent TB. A recent review of the status of anti-TB drugs is provided by Hickey et al. [61]. Small clinical trials suggest that immunotherapy with inhaled interferon-gamma [62], or inhalation of a dry powder formulation of the antibiotic capreomycin with a hand-held inhaler (Cyclohaler^®^, Plastiape, Italy), might be beneficial to TB patients [63]. However, large, randomized controlled clinical trials are needed to further evaluate efficacy and safety.

Mucosal immunity for protection against TB has been theorized, but is yet unproven in clinical trials. Nevertheless, a number of novel formulations including nanoparticles and dry powders of antigen/adjuvant combinations are being evaluated in animal models [64]. Two examples are provided here. A suspension of nanoparticles (i.e. particles <0.1 μm in diameter) conjugated with Ag85B tuberculosis antigen and delivered through the nostrils of mice showed better protection against subsequent challenge, compared to intradermal delivery [65]. A dry powder of live- attenuated tuberculosis vaccine bacille Calmette-Guerin (BCG), administered by an Insufflator^®^, resulted in a significantly reduced bacterial burden and lung pathology in guinea pigs subsequently challenged with virulent Mycobacterium tuberculosis, compared to untreated animals and control animals immunized with the standard parenteral BCG [66]. Further investigation is needed to bring these products forward.

Immunization against the human papilloma virus by inhalation has been tested in a small clinical trial by Nardelli-Haefliger and colleagues [67]. This was a dose escalation study of intranasal and oral inhalation of a human papilloma virus-like particle (HPV16 VLP) vaccine aerosol. Nasal administration was via a Devilbiss^®^ nebulizer sprayed into each nostril. Pulmonary administration was achieved using a sonication-type nebulizer and mouthpiece. Healthy adult female volunteers inhaled two doses of the vaccine on day 0 and day 2 by nose, or mouth. Doses escalated from 2 μg to 50 μg and 250 μg. Volunteers who inhaled 250 μg by mouth seroconverted (an indicator of vaccination) and the magnitude of their serum IgG and IgA responses was similar to that seen with an historically-treated group that was administered 50 μg by intramuscular injection. Lower doses by oral inhalation were less effective and intranasal vaccination was poorly immunogenic for most volunteers. These data raise the possibility that administration of the VLP vaccine via oral inhalation may offer an alternative to systemic immunization. More trials are needed to confirm that aerosol vaccination is safe, immunogenic and protective against genital HPV infection.

Gordon et al. [68] compared the effect of intramuscular vs. inhaled 23-valent pneumococcal capsular polysaccharide vaccine (23-PPV) on pulmonary mucosal immunoglobulin levels. Vaccine was delivered by jet nebulizer (Sidestream^®^, Respironics, Murrysville, PA, USA). Bronchoalveolar lavage (BAL) and serum were collected from 33 adults before and 1 month after injected (n=16) or inhaled (n=17) 23-PPV. Levels of pneumococcal capsule-specific IgG and IgA to types 1, 9V and 14 were measured in each sample. Injected 23-PPV produced a significant increase in types 1, 9V and 14 capsule-specific IgG and type 1 IgA in both serum and BAL. Inhaled vaccine produced no response in either BAL or serum.

#### Best example of vaccination by inhalation: preventing measles with inhaled measles vaccine

Inhaled measles vaccine is furthest along in drug development, compared to the other inhaled vaccine candidates mentioned above and is, therefore, the best example of vaccination by inhalation. Its development has also been greatly influenced by lessons learned from the Exubera^®^ inhaled insulin experience. The worldwide incidence of measles has been declining for the last ten years. However, populations in some countries remain unprotected. For example, an estimated 20 million children worldwide were under-vaccinated in 2012 [69]. If infants are not adequately immunized against measles, the entire community will be at risk for measles epidemics.

Over three decades ago, Dr. Albert Sabin and colleagues proved the feasibility of vaccination by aerosolized measles vaccine [70, 71]. Since then, other trials have demonstrated that measles vaccine administered by aerosol provides a superior boosting response compared to vaccination by injection in school-age children [72, 73]. However, studies performed in infants younger than 10 months of age showed that seroconversion rates were lower with aerosolized than subcutaneous vaccine [74]. This may have been due to an inadequate dose delivered to the lungs of these infants, since an inefficient nebulizer and face mask was used in those trials. Thus, a major challenge to developing an aerosolized vaccine for preventing measles in the developing world is to deliver an adequate dose to infants.

A second challenge to the development of an inhalable measles vaccine is the need for new delivery devices. Such delivery devices need to be efficient, portable and battery operated, since electricity is not readily available in villages in developing countries. New, efficient, portable devices that are battery-operated are now available for liquid aerosol deliver (i.e. vibrating mesh devices) and for dry powder delivery. Details about how these devices operate and their proper use can be found elsewhere [5]. However, it is only within the last 4–5 years that these devices have been incorporated into clinical trials that are testing the efficacy of inhalable measles vaccine. Unfortunately, many of these trials have only recently been completed, or are being completed, and results are not yet published. An update of the status of those trials, the formulation of measles vaccine being tested and the devices that are being used to deliver the vaccine is provided below.

**Figure 5:**
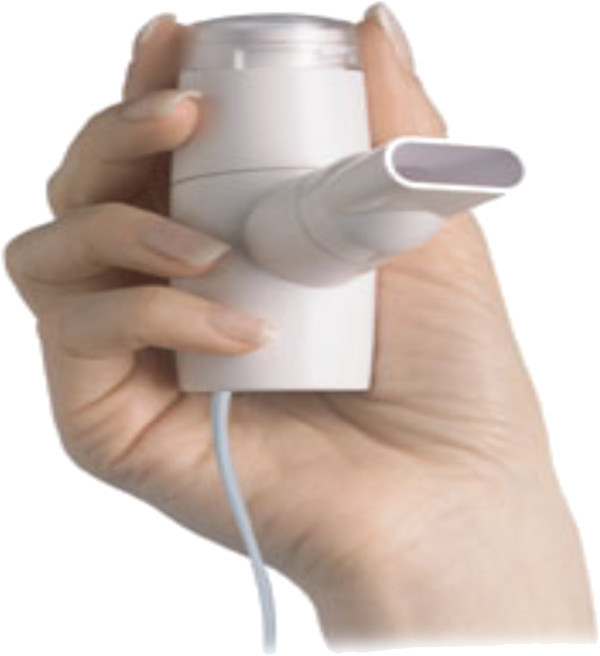
**The Aerogen Aeroneb Go** ^**®**^ **delivery system (Aerogen, Galway Ireland) is being developed to deliver liquid measles vaccine to children and infants in developing countries.** The device consists of a medication chamber that is located in the upper part of a plastic holder. The outlet port extends from the holder and can be fitted with a mouthpiece or facemask. A vibrating membrane, the generator OnQ^®^, pumps fluid through small holes generating aerosol with a median diameter of 3.6 microns. The control module works with three AA batteries and is connected to the medication chamber through a removable cord [75]. (Image downloaded from Aerogen website at http:www.aerogen.com/aeroneb-go.html).

In the early 2000s, the World Health Organization (WHO) began work on an aerosolized measles vaccine that could be used in mass immunization campaigns in developing countries. The WHO decided to aerosolize the liquid formulation that was licensed for injection therapy and had proven effective by inhalation in earlier studies in Mexico [70, 71]. This was the Edmonston-Zagreb (EZ) live-attenuated measles vaccine. This choice meant that the WHO did not have to reformulate the vaccine, which could have resulted in years of additional testing. After several years of device development in collaboration with the U.S. CDC, the Bill and Melinda Gates Foundation and Aerogen (Galway, Ireland), the WHO began testing the Aerogen AeronebGo^®^ delivery system in India. This is a portable, battery-operated vibrating mesh device with a face mask for infant aerosol delivery (Figure 5). A Phase III trial in 2,000 children <12 months old was recently completed. Data are being analyzed and results will be compared to those obtained with subcutaneous (SC) administration of measles vaccine in a similar age-group.

The possibility of delivering a combination aerosol vaccine to protect against measles, mumps and rubella with the AeronebGo^®^ device has also being conducted [75]. This was an exploratory study to evaluate the safety and antibody responses to each component of MMR II (Attenuvax measles live-attenuated vaccine, Jeryl Lynn mumps live-attenuated vaccine and L-Zagreb mumps live-attenuated vaccine) in healthy adults 21–38 years of age. The investigators chose to use the AeronebGo^®^ device because previous studies with aerosolized Schwarz measles vaccine (similar to the Attenuvax base sequence) showed rapid degradation of vaccine potency using a jet nebulizer and compressed air system. Results from the more recent study showed that aerosolization of the three components of MMRII vaccine was safe and produced secondary immune responses in healthy adults. Similar safety studies need to be performed in children.

**Figure 6:**
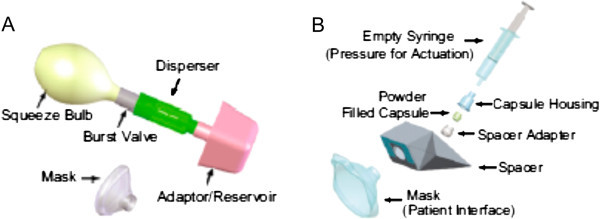
**The PuffHaler** ^**®**^ **(Aktiv-Dry, LLC, Boulder, CO, USA) (A) and the Solovent** ^**®**^ **(Becton, Dickinson and Company, Franklin Lakes, NJ, USA) (B) are two new devices that are being developed to deliver measles vaccine as a dry powder to children and infants in developing countries.** When the PuffHaler squeeze bulb is compressed, the silicone rubber burst-valve pops open. The air rushes into the disperser through the powder in an aluminum foil blister and the aerosol cloud fills a collapsed plastic bag reservoir. The aerosol-filled bag is detached and affixed to a facemask from which the subject breathes for 30 s to become vaccinated (Puff-mask). The syringe of the BD Solovent device is used to pressurize the capsule containing the powder vaccine. As the pressure rises, the thin films sealing the capsule rupture, and the powder is expelled and captured in the disposable spacer for delivery through a silicone facemask (Sol-mask) (from Reference [76] with Permission).

Within the last 6–8 years, the WHO, the U.S. CDC, the Bill and Melinda Gates Foundation, the NIH and Aktiv-Dry, LLC (Boulder, CO, USA) began working on a powder formulation of the EZ live-attenuated measles vaccine. The advantage to a powder formulation is that it does not need refrigeration, which is also often lacking at sites of mass campaigns in developing countries. The powder was developed by Aktiv-Dry, LLC. The two devices to deliver the powder are called the PuffHaler^®^ and the Solovent^®^ and are shown in Figure 6A and 6B. They were developed by Aktiv-Dry and BD Technologies (Becton, Dickinson and Company), respectively. Both devices incorporate a holding chamber that allows the powder to be actuated and held in place until inhalation is initiated. Such a holding chamber for a powder aerosol was first introduced in the Exubera^®^ Pulmonary Insulin Delivery System that was used to administer insulin aerosol. Unlike the Exubera device, these devices are constructed of inexpensive materials such that the cost of delivering dry powder measles vaccine will not cost more than intramuscular injection administration. Both devices also include flexible face masks for infant delivery. In a recent pre-clinical trial, dry powder vaccine delivered by these two devices provided full protection against measles infection in Rhesus macaques [76]. A large clinical trial to test the efficacy of this powder formulation in humans is currently being planned.

Unlike aerosol applications for systemic drug delivery and gene therapy, immunization by inhalation does not require chronic repeat dosing for efficacy. For many vaccines, immunization may be achieved with 1–2 aerosol treatments followed by a booster treatment. Thus, there is less concern about the safety of repeated lung dosing with immunization by inhalation, compared to systemic drug delivery and gene therapy by inhalation. Nevertheless, safety remains an issue with inhaled vaccines because some patient populations (e.g. patients with allergic asthma) may be more sensitive to excipients in the formulations and care-givers who are immunosuppressed may be more vulnerable to vaccine exposure than non-immunosuppressed individuals.

#### Future directions

Vaccination by inhalation is a promising new method for immunization. It has already been used in large populations and appears to be a feasible method for mass vaccinations. Recent developments in device innovation have made reliable, portable aerosol dosing in mass campaigns possible. Improvements in delivery to infants and in the development of vaccines that do not require refrigeration (i.e. powders) and are stable at the ambient temperatures of the tropics could make this the preferred route of administration for a number of vaccines in the future.

## Conclusions

The role of aerosol therapy has changed over the years to now include systemic drug delivery by inhalation, inhaled gene therapy and vaccination by inhalation. Each of these new applications has led to the development of new delivery devices and achieved varying degrees of success in treating their disease targets. The continued expansion of the role of aerosol therapy in the future will depend on: (1) improving the bioavailability of systemically delivered drugs; (2) developing gene therapy vectors that can efficiently penetrate the airway mucus barrier and cell membrane, navigate the cell cytoplasm and efficiently transfer DNA material to the cell nucleus; (3) improving delivery of gene vectors and vaccines to infants; and (4) developing formulations that are safe for acute and chronic administrations.
